# Hemmorrhage Arrested

**Published:** 1873-10

**Authors:** N. D. Lewis


### HEMMORRHAGE ARRESTED.

BY N. D. LEWIS.

Not long since I had a root extracted from the upper jaw, which bled most of the time for two days and nights. I could not stop the bleeding with the usual remedies, I tried kerosene oil by wetting a small plugget of cotton and inserting it in the cavity. The bleeding stopped in a few minutes and healing took place without sloughing, or other bad effects.

